# Influence of Different Grades of CBN Inserts on Cutting Force and Surface Roughness of AISI H13 Die Tool Steel during Hard Turning Operation

**DOI:** 10.3390/ma12010177

**Published:** 2019-01-07

**Authors:** Pardeep Kumar, Sant Ram Chauhan, Catalin Iulian Pruncu, Munish Kumar Gupta, Danil Yurievich Pimenov, Mozammel Mia, Harjot Singh Gill

**Affiliations:** 1 Department of Mechanical Engineering, Deenbandhu Chhotu Ram University of Science and Technology, Murthal, Haryana 131039, India; psharma.dcrust@gmail.com; 2 Department of Mechanical Engineering, National Institute of Technology, Hamirpur (H.P.) 177005, India; santramchauhan@gmail.com; 3 Mechanical Engineering, Imperial College London, Exhibition Rd., London SW7 2AZ, UK; 4 Mechanical Engineering, School of Engineering, University of Birmingham, Birmingham B15 2TT, UK; 5 Department of Mechanical Engineering, Chandigarh University, Gharuan, Punjab 140413, India; munishguptanit@gmail.com (M.K.G.); harjot.gill@cumail.in (H.S.G.); 6 Department of Automated Mechanical Engineering, South Ural State University, Lenin Prosp. 76, Chelyabinsk 454080, Russia; danil_u@rambler.ru; 7 Mechanical and Production Engineering, Ahsanullah University of Science and Technology, Dhaka 1208, Bangladesh; mozammelmiaipe@gmail.com

**Keywords:** CBN, cutting forces, optimization, turning, surface roughness

## Abstract

Now-a-days, the application of hard tuning with CBN tool has been massively increased because the hard turning is a good alternative to grinding process. However, there are some issues that need to be addressed related to the CBN grades and their particular applications in the area of hard turning process. This experimental study investigated the effects of three different grades of CBN insert on the cutting forces and surface roughness. The process of hard turning was made using the AISI H13 die tool steel at containing different hardness (45 HRC, 50 HRC and 55 HRC) levels. The work material were selected on the basis of its application in the die making industries in a range of hardness of 45–55 HRC. Optimization by the central composite design approach has been used for design and analysis. The present study reported that the cutting forces and surface roughness are influenced by the alloying elements and percentage of CBN in the cutting tool material. The work material hardness, feed rate and cutting speed are found to be statistically significant on the responses. Furthermore, a comparative performance between the three different grades of CBN inserts has been shown on the cutting forces and surface roughness at different workpiece hardness. To obtain the optimum parameters from multiple responses, desirability approach has been used. The novelty/robustness of the present study is represented by its great contribution to solve practical industrial application when is developed a new process using different CBN grades for hard turning and die makers of workpiece having the hardness between 45 and 55 HRC.

## 1. Introduction

Manufacturing industries continue to require superior cutting tools that are capable to generate tight tolerance and economical material production. Particularly in the area of hard machining, selection of cutting tool always remains a challenging task for the manufacturer. The various cutting tools such as carbide, ceramic, cubic boron nitride (CBN) and poly crystalline diamond (PCD) are being used and established successfully for the hard machining process, however, the CBN tool is known to be the best choice for hard machining, because of its higher thermal conductivity and hardness among carbide and ceramic tools [1]. Although, the PCD has a higher hardness in respect to CBN tool, its chemical reactivity with ferrous material makes it unsuitable for hard machining. Therefore, the use of CBN is known to be the best choice for hard turning operation. In 1972, CBN was first developed with cobalt as a binder, and a subsequent advancement in the cutting tool technology were made. The CBN tools are broadly classified as High-CBN and Low-CBN; these classifications are made on the basis of percentage of CBN content and the alloying elements or binders such as cobalt, ceramic, TiC, TiN etc. in the CBN matrix. These binders and alloying elements, directly affects the various properties of the CBN tool material such as thermal conductivity, hardness, rupture strength etc. Therefore, before selection of CBN grade, the prior knowledge of its various properties is highly required to be identified, because different grades are suitable for different machining applications such as in interrupted cutting, continuous cutting etc. 

In the past two decades, a large amount of work has been done by the researchers in the field of turning process made of CBN cutting tools to improve the manufacturability. Koing et al. [2] proves the consequences of binders in CBN tools, and found that binder’s composition and percentage affects the thermal stability of the CBN tools. Similarly, Eda et al. [3] shows that bond strength of a CBN grain with metallic binders is lesser than the bond strength of CBN grains with ceramic binders. Therefore, the ceramic binders based on CBN have lesser toughness. Likewise, Bossom et al. [4] studied the comparisons of thermal conductivity of CBN-H and CBN-L tools, and found that CBN-H possess higher thermal conductivity. Some, study also revealed that CBN-L shows better performance during finish turning when compared to CBN-H. In another work, Bushlya et al. [5] investigated the performance of coated and uncoated CBN tools. They found that the coated tools produce higher cutting force when compared to uncoated CBN tools. Huang et al. [6] studied the thermal properties of CBN tool on cutting forces during hard turning of AISI H13 steel. The results revealed that High-CBN tool produces higher cutting forces as compare to Low-CBN inserts. Therefore, seems that it is necessary to select an appropriate grade of CBN cutting tool for a proper and economic hard machining operation. 

### Motivation

From the state of art review, it has been shown that the application of cubic boron nitride (CBN) tools make hard machining process promising in the industrial field, and the last two-decade was demonstrated that hard machining was an important research area in manufacturing fields. Hard machining also known as finish machining, is an emerging process that enables the manufacturers to machine material having hardness more than 45 HRC. The important benefits of this process are reducing the product cost, lead time and number of setup changes, without compromising surface quality [7]. There are great demands of hardened steel in various applications like, cutting tools, thread rolls, burnishing rolls and in die making industries. AISI H13 is a hot working die tool steel and is widely used for making forging, extrusion dies. Generally, the material used for making dies is in the hardness range of 45–65 HRC. The machining at higher hardness led to a higher cutting force as compared to the conventional turning process. It has been reported that during the turning of a material at 55 HRC, the forces are 30% higher than the turning of the similar material in the annealing conditions [8]. Therefore, the cutting forces are the major concern during hard turning as they are associated to the various cutting performance (like tool wear [9,10], vibrations and accuracy of the machined surface [11,12,13,14], surface topography [15,16]) and design of machine elements, fixtures, tool holders etc. In the die and mould maker’s industry, the surface finish is the major concern. The hard-turning process can generate the average surface roughness parameter less than 0.3µm in a single set-up and the process can maintain a size tolerance of 0.010 mm or better [8]. The CBN tools may generate surface roughness values that are comparable to the grinding process. Further, the high-CBN tools produce damage free surface (surface integrity) as compared to the low-CBN tools [17]. The tool wear increases with the increase in cutting speed for each higher grade of CBN inserts. During hard turning the surface roughness values were observed to increase from 0.2 μm to 0.6 μm, and it increases with the increase in the feed rate [18]. Other studies revealed that the feed rate and cutting-edge geometry had a great impact on the surface quality of turned parts. It has also been observed that surface roughness increases with the increase in hone edge radius because of the ploughing effects [19]. Superior performance is obtained on the hard turning using a minimal cutting fluid application in comparison to dry turning and wet conditions [20]. Feng et al. [21] developed an empirical model for surface roughness to study the influences of cutting speed, feed rate, depth of cut, cutting time and workpiece hardness during hard turning. Design of experiment and neural network approach were used to optimize the influence of cutting speed, feed rate and depth of cut on flank wear [22]. In conclusion, a non-robust agreement was made regarding the optimum condition when turning hard turning operation. As hard turning is known for its precise machining, therefore, a proper selection of cutting tools must be developed. Although CBN is best known tool in the area of hard turning, still there is a need of investigation of CBN grades in the area of hard turning so that the process can be used at its full potential. Therefore, present study investigates the effects of three different grades of CBN tools during hard turning operation of AISI H13 work material with three different hardness. An overview comparison between the surface roughness and cutting forces generated by the different grades of CBN tools were developed.

## 2. Materials and Methods

### 2.1. Work Material

In the present work, the AISI H-13 hot work die tool steel has been selected on the basis of its wide applications in the moulds and die making industries, having a hardness range of 45 HRC to 60 HRC. The material selected withstand to high mechanical and thermal stresses. Three work specimens with a hardness of 45 HRC, 50 HRC and 55 HRC (±1 HRC) have been used for the hard-turning experiments. Specimens were used in the form of round bars having 50 mm diameter and 150 mm length. The workpiece was thoroughly hardened by tempering process to attain the respective hardness. The chemical composition of the work material has been shown in Table 1.

### 2.2. Cutting Tool

**Figure 1:**
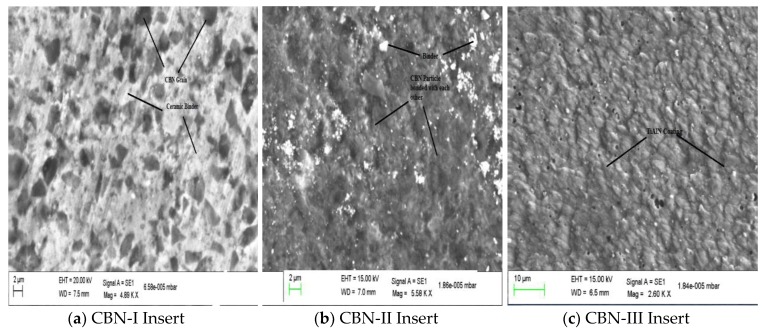
SEM images patterns of different CBN inserts.

Based on different properties and CBN composition (%), three different grades of CBN tools have been selected for their comparative performance during hard turning of AISI H13 die tool steel. These grades are BNX-10, BN-600 and BNC-300 and were designated in the study as CBN-I, CBN-II and CBN-III, respectively. These grades convey to SUMITOMO company make (Japan) and their properties are shown in Table 2. Figure 1 shows the SEM images of different grades of CBN insert. From the SEM images it can be noticed that CBN-I has the lesser amount of CBN percentage as compare to CBN-II insert, while CBN-III is coated with TiAlN coating. Inserts geometry are prepared as per ISO standard A-40 which can be classified as follows: inserts type CCMT 09T308 chamfered edged (30° × 0.1 mm for CBN-I and CBN-II and 25° × 0.05 mm for CBN-III) geometry has been selected of rake angle 10°, nose radius = 0.8 mm, and relief angle = 7°.

### 2.3. Machine Set-Up and Measurements

**Figure 2:**
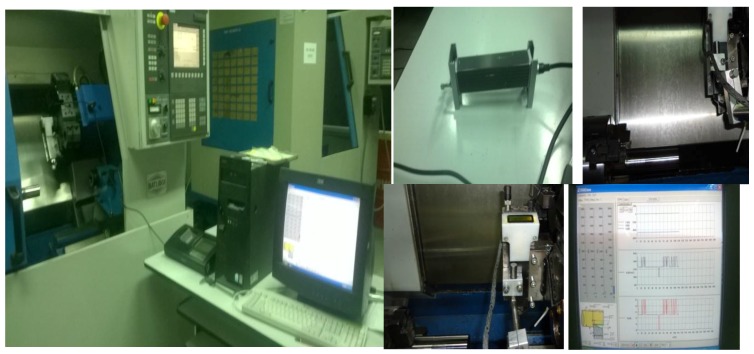
Experimental Set-up.

Hard turning experiments were performed on a SPRINT 16 TC CNC (BATLIBOI; Mumbai, India) machine as shown in Figure 2. All the experiments were performed under dry machining condition and all the measurement are done in a laboratory condition at a temperature of 26 °C and humidity of 34% according to reference standards. A Mitutoyo make (SJ-301) surface roughness tester (Mitutoyo, Japan) has been used to measure the surface roughness. The ISO 4287:2000 standard has been used to measure the surface roughness which is based on the mean line system. In present work, DKM2010 turning dynamometer (TeLC, Unna, Germany) were used. DKM2010 is a 5-components tool dynamometer for use on every type of lathe machine be conventional or CNC. It allows to measure the forces on the cutting tool up to 2000 N with a resolution of 0.1%, hence here it was used to measure the cutting forces (*F_c_* and *F_t_*). The dynamometer in conjunction with XKM 2000 software (TeLC, Unna, Germany) was used, along with a personal computer (PC) to transfer the data acquisition as shown in Figure 2. 

### 2.4. Design of Experiments

The objective of this experimental study were to find out the comparative performance of the three different grades of CBN tool on the tangential force (Fc), thrust force (Ft) and surface roughness (Ra) during hard turning of AISI H13 die tool steel. For the above investigation, four parameters such as cutting speed (A), feed rate (B), depth of cut (C) and workpiece hardness (D) have been selected, Table 3 illustrates the ranges of the respective parameter. Moreover, this experimental investigation modelled and found the optimum formulation for the selected responses; hence, the response surface methodology (RSM) technique has been used for design of experiments and analysis of results. RSM is generated as an interaction between statistical and mathematical techniques, which is used to model and analyze the response variables that are influenced by several variables [23]. RSM permits to establish the relationship between one or more response variable and essential controllable input variables [24]. The experiments were designed on the basis of rotatable central composite design (CCD), in which the radial distance of all the points are equidistance from the centre point. CCD is generally used for the modelling of second order response, and is most commonly used method in the RSM [25]. On the basis of this design of experiment, with four factors and three levels, a total of 30 runs with 6 centre points were employed for each CBN inserts. Table 4 represents the respective responses according to the design matrix.

## 3. Results

To check the significance and adequacy of the developed models, various tests have been performed such as significance test, lack of fit test and individual model coefficient test. ANOVA tables summarized the model adequacy for each response. ANOVA table represents the model terms significant, for a value in which “Prob. > F” is less than 5%. Coefficient of variance (CV) and correlation (R2) check the precision, reliability and variability of the results. Prediction error sum of square (PRESS) assess that how the present model is compatible with the new experiments where always a small value of PRESS is desirable [26,27]. For statistical analysis and design of experiments, design expert (DX-7) strategy has been used as per RSM methodology. 

### 3.1. Analysis for Surface Roughness (Ra) 

A Quadratic model has been implemented for investigated the surface roughness for CBN-I, CBN-II and CBN-III inserts. Table 5, Table 6 and Table 7 presents all the significant model terms in the respective table. Table 5 shows the ANOVA results for CBN-I insert, in which the model is found to be significant as the model “F-value” was 12.01. Table 5 illustrate that for the quadratic model, A, B, C and D model term were found to be significant whereas “lack of fit” for the value of 4.30 is found insignificant. Similarly, Table 6 and Table 7 illustrate the ANOVA results for the inserts CBN-II and CBN-III, respectively. Table 6 and Table 7 also shows the significant quadratic model and insignificant lack of fit.

#### 3.1.1. Responses Surface Model for Surface Roughness (Ra) 

Experimental model developed by the CBN-I, CBN-II and CBN-III for surface roughness are as follows:

(1) $$\begin{array}{ll} Surface & Roughness_{\left(\mathrm{CBN-I}\right)}\\ & =0.46-0.086\times A+0.17\;\times B+0.061\times C-0.18\times D-0.024\times A\\ & \times B-0.015\times A\times C+0.019\times A\times D+0.0002\times B\times C-0.036\\ & \times B\times D-0.027\times C\times D+0.012\times A^{2}-0.058\times B^{2}+0.027\times C^{2}\\ & +0.097\times D^{2} \end{array}$$

(2) $$\begin{array}{ll} Surface & Roughness_{\left(\mathrm{CBN-II}\right)}\\ & =0.53-0.093\times A+0.24\times B+0.065\times C-0.26\times D-0.026\times A\\ & \times B-1.000E-002\times A\times C+8.750E-003\times A\times D+0.019\times B\\ & \times C-0.13\times B\times D-0.044\times C\times D-0.026\times A^{2}-0.011\times B^{2}\\ & +0.069\times C^{2}+0.11\times D^{2} \end{array}$$

(3) $$\begin{array}{ll} Surface & Roughness_{\left(\mathrm{CBN-III}\right)}\\ & =0.68-0.13\times A+0.29\times B+0.039\times C-0.31\times D-0.028\times A\\ & \times B+6.250E-003\times A\times C+0.021\times A\times D-0.010\times B\times C\\ & -0.16\times B\times D-0.021\times C\times D-0.019\times A^{2}-0.044\times B^{2}+0.086\\ & \times C^{2}+0.11\times D^{2} \end{array}$$

#### 3.1.2. Parametric Influence on Surface Roughness (Ra)

**Figure 3:**
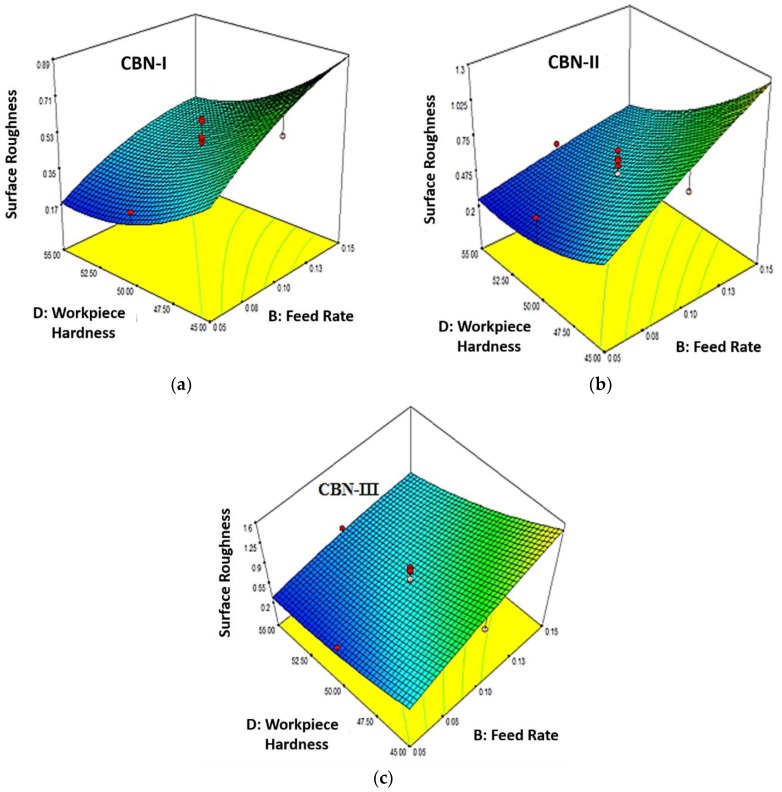
(**a**) Effect of Feed Rate (B) and Work Material Hardness (D) on Surface Roughness (R_a_) for CBN-I insert (**b**) Effect of Feed Rate (B) and Work Material Hardness (D) on Surface Roughness (R_a_) for CBN-II insert (**c**) Effect of Feed Rate (B) and Work Material Hardness (D) on Surface Roughness (R_a_) for CBN-III insert.

Figure 3a–c illustrates the consequences of feed rate and workpiece hardness on surface roughness for three different inserts as CBN-I, CBN-II and CBN-III, respectively. From the figures it seems that surface roughness decreases with the increase in the workpiece hardness. It can be seen that the workpiece with a higher hardness (55 HRC) produce a better surface roughness as compared to lower workpiece hardness (45 HRC and 50 HRC). These results can be explained as: with the increases in the workpiece hardness the plasticity of the material decreases therefore lateral plastic flow decreases, hence there is an improvement in the surface roughness. Chavoshi et al. [28] reported a similar trend when the increase in the workpiece hardness was noted, there the surface roughness decreases. The figures also show that surface roughness increases with the increase in feed rate for different CBN inserts. Similar evidence of changes in the roughness are noted in the works of Abbas et al. [29,30,31]. As feed rate increases, that results increase in undeformed chip thickness, and undeformed chip thickness is directly proportional to cutting force. Therefore, if cutting force increases it will affects the stability and damping characteristics, which cause vibration and ultimately affects the surface roughness of the surface. 

**Figure 4:**
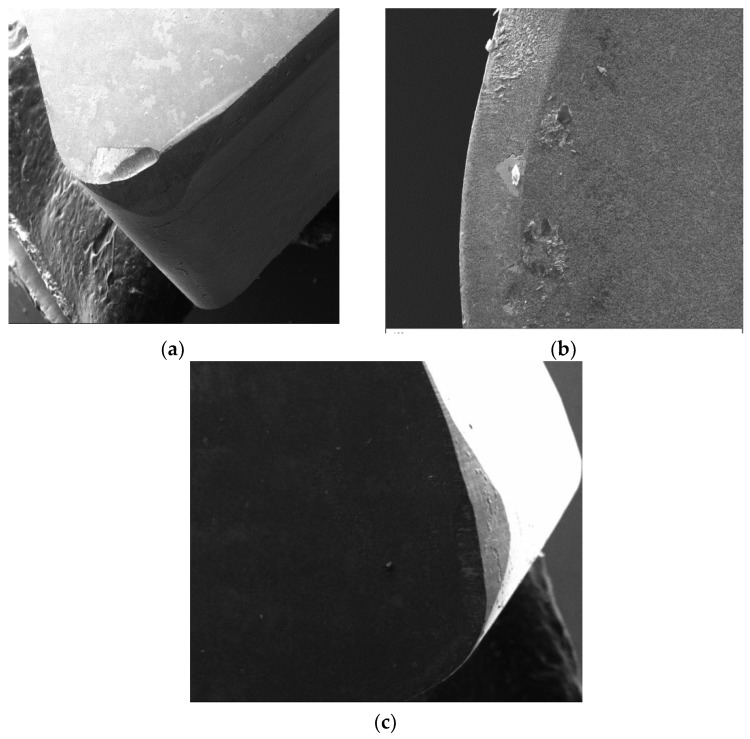
Tool wear images patterns (**a**) CBN-I (**b**) CBN-II (**c**) CBN-III highlighting the abrasion arks on rake face and Flank Wear on Flank Face.

Further, the surface roughness can be compared with the tool wear of different grades of CBN–I, CBN-II& CBN-III inserts as shown in Figure 4a–c, respectively. It can be observed from the given Figure 4b that, the CBN-II inserts wear out due to the erosion of the CBN particle form the CBN matrix. CBN-II tools are the metallic binders-based substrate, having lower bonding strength. Due to the lower strength between CBN particles and binders, CBN particles plucked out from the substrate. These hard CBN particles having hardness of around 2800-4200HV are the main cause of abrasive wear on the tool surface [32]. CBN-I have lower thermal conductivity therefore more heat dissipated into the work material which cause the thermal softening in the work material at the shear zone. Figure 3a depicts the SEM image of low-CBN tool. Figure 4c shows the tool wear images of CBN-III tool, as CBN-III generates the higher cutting forces results in poor surface finishing. 

**Figure 5:**
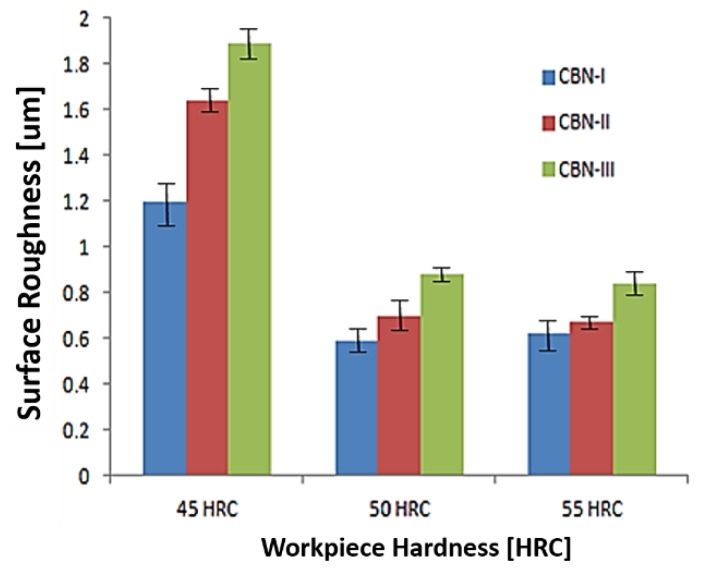
Comparisons of Surface roughness obtained with different CBN insert corresponding to workpiece hardness.

Figure 5 shows the comparison of maximum surface roughness for different workpiece hardness with CBN-I, CBN-II and CBN-III inserts. Figure 5 illustrate that CBN-I insert generate better surface roughness for different workpiece hardness in comparison with other two CBN inserts. CBN-I generate the surface roughness of value in a range of 0.37–1.2 μm, 0.24–0.59 μm and 0.14–0.62 μm corresponding to 45 HRC, 50 HRC and 55 HRC, respectively. This can be explained in terms of CBN-I having poor thermal conductivity as compared to CBN-III, while CBN-II is known for its superior thermal conductivity. Therefore, more heat dissipated into work material during hard turning of AISI H13 die tool steel with CBN-I insert that cause thermal softening effects on shear plane region. Aouici et al. reported the behavior of thermal softening of the material during machining [33]. 

### 3.2. Analysis for Tangential Forces (Fc)

Quadratic model has been developed do detect the tangential force for all the three CBN inserts. Table 8, Table 9 and Table 10 represent the ANOVA results for CBN-I, CBN-II and CBN-III inserts, respectively. Table 8 shows that quadratic model is significant for the “*F*-value” of 34.94, and found insignificant lack of fit for the value of 0.93. The model illustrate that A, B, C, D, BC and BD are the significant model terms. Similarly, Table 9 and Table 10 represent the significant model for CBN-II and CBN-III inserts, respectively. 

#### 3.2.1. Response Surface Model for Tangential Force (Fc)

Experimental model developed by the CBN-I, CBN-II and CBN-III for tangential force (*Fc*) is as follows:

(4) $$\begin{array}{ll} Tangential & Force_{\left(\mathrm{CBN-I}\right)}\\ & =75.96-2.44\times A+8.56\times B+8.61\times C+14.17\times D-0.37\times A\\ & \times B+0.50\times A\times C+0.000\times A\times D-2.50\times B\times C-4.50\times B\times D\\ & +1.63\times C\times D-1.60\times A^{2}-3.60\times B^{2}-5.10\times C^{2}-1.10\times D^{2} \end{array}$$

(5) $$\begin{array}{ll} Tangential & Force_{\left(\mathrm{CBN-II}\right)}\\ & =79.58-3.33\times A+10.33\times B+9.22\times C+13.39\times D+0.56\times A\\ & \times B+1.69\times A\times C-0.69\times A\times D-0.19\times B\times C-3.56\times B\times D\\ & +0.56\times C\times D-3.49\times A^{2}-2.49\times B^{2}-3.49\times C^{2}+8.772E\\ & -003\times D^{2} \end{array}$$

(6) $$\begin{array}{ll} Tangential & Force_{\left(\mathrm{CBN-III}\right)}\\ & =87.18-3.61\times A+9.67\times B+11.00\times C+14.28\times D-0.75\times A\\ & \times B+1.00\times A\times C-3.37\times A\times D-2.12\times B\times C-2.75\times B\times D\\ & +0.75\times C\times D-6.85\times A^{2}-2.35\times B^{2}-1.35\times C^{2}+7.15\times D^{2} \end{array}$$

#### 3.2.2. Parametric Influence on Tangential Force (Fc)

**Figure 6:**
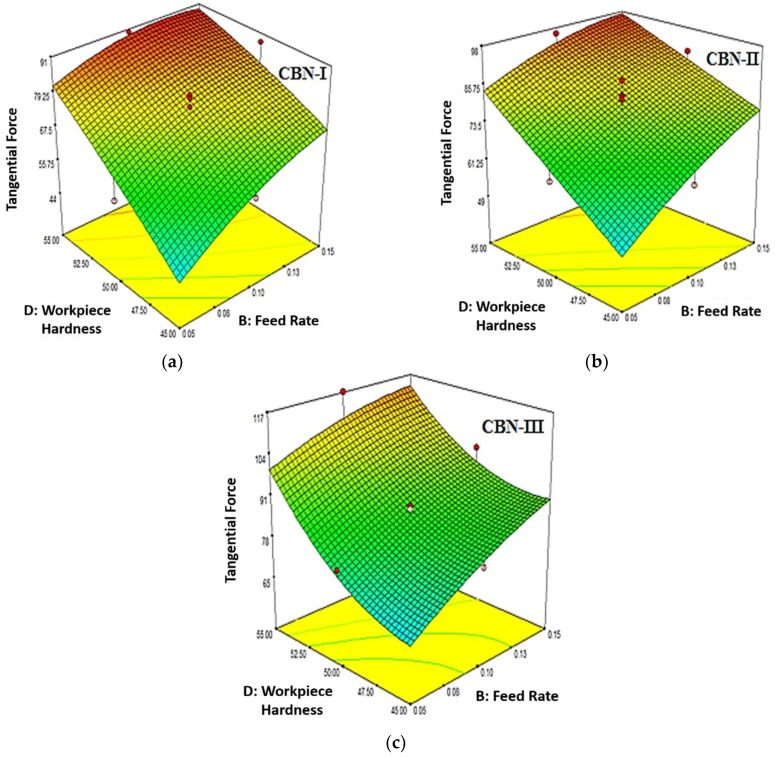
(**a**) Effect of Work Material Hardness (D) and Feed Rate (B) on Tangential Force (F_c_) for CBN-I insert; (**b**) Effect of Work Material Hardness (D) and Feed Rate (B) on Tangential Force (F_c_) for CBN-II insert; (**c**) Effect of Work Material Hardness (D) and Feed Rate (B) on Tangential Force (F_c_) for CBN-III insert.

Figure 6a–c revealed the variation of tangential force corresponding to feed rate and workpiece hardness for CBN-I, CBN-II and CBN-III inserts, respectively. The Figure 6 shows that tangential force increases with increase in feed rate and workpiece hardness of the material. Higher work material hardness 55 HRC exhibit maximum tangential force when compared to material hardness of 45 HRC and 50 HRC. This can be explained by the higher hardness that can have fine grain structure as compared to lower hardness, therefore higher force is required to deform the material. Figure 6a–c also illustrate that tangential force increases with the increase of feed rate. The increment in the feed rate cause increase in undeformed chip thickness, as a result more force will be required. Moreover, a high feed rate is explicitly associated with the tool geometry, especially the nose radius. When the nose radius is greater, a higher stress is developed on the tool tip. Conversely, it can be claimed that the cutting force was increased in such cases.

**Figure 7:**
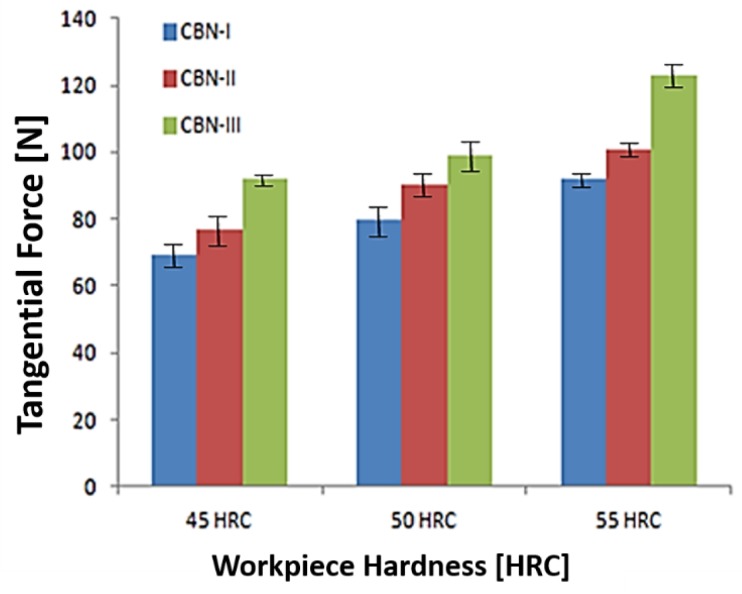
Comparisons of Tangential Force (F_c_) with different CBN insert corresponding to workpiece Hardness.

Figure 7 depicts results of CBN-III insert that exhibit higher maximum tangential force when compared to CBN-II and CBN-I inserts. It has also been observed that tangential force increases with the increase in material hardness. CBN-III generates the tangential force in the range of 44–92 N, 77–99 N and 67–123 N corresponding to 45 HRC, 50 HRC and 55 HRC, respectively. While tangential force was found to be minimum in a range of 26–69 N, 56–80 N and 61–92 N corresponding to material hardness of 45 HRC, 50 HRC and 55 HRC with CBN-I insert. This can be explained in terms of the hardness of different CBN inserts, and CBN-III that exhibit the higher hardness compare to other two inserts. 

### 3.3. Analysis for Thrust Force (Ft)

ANOVA results for thrust force have been illustrated in Table 11, Table 12 and Table 13 for CBN-I, CBN-II and CBN-III inserts, respectively. Table 11 shows the ANOVA results of thrust force for CBN-I insert, in which quadratic model was found to be significant for the “F-value” of 15.06 and a non-significant, lack of fit, for the value of 4.33. In the quadratic model B, C, D, CD and C2 are the significant model terms. Similarly, Table 12 and Table 13 illustrate the significant quadratic model for CBN-II and CBN-III inserts. 

#### 3.3.1. Response Surface Model for Thrust Force (Ft)

Experimental model developed by the CBN-I, CBN-II and CBN-III for thrust force (Ft) are as follows:

(7) $$\begin{array}{ll} Thrust & Force_{\left(\mathrm{CBN-I}\right)}\\ & =116.51-4.50\times A+13.06\times B+17.61\times C+26.50\times D+2.06\times A\\ & \times B-1.44\times A\times C-2.06\times A\times D-1.19\times B\times C-5.06\times B\times D\\ & +9.44\times C\times D+0.48\times A^{2}+3.48\times B^{2}-14.52\times C^{2}+4.48\times D^{2} \end{array}$$

(8) $$\begin{array}{ll} Thrust & Force_{\left(\mathrm{CBN-II}\right)}\\ & =121.90-6.67\times A+12.56\times B+19.17\times C+27.11\times D+0.000\times A\\ & \times B-0.62\times A\times C-2.37\times A\times D-1.37\times B\times C-5.12\times B\times D\\ & +7.50\times C\times D-3.31\times A^{2}+1.69\times B^{2}-8.81\times C^{2}+6.69\times D^{2} \end{array}$$

(9) $$\begin{array}{ll} Thrust & Force_{\left(\mathrm{CBN-III}\right)}\\ & =132.00-5.06\times A+13.06\times B+20.67\times C+25.83\times D+0.19\times A\\ & \times B+1.94\times A\times C-2.69\times A\times D+1.06\;\times B\times C-7.31\times B\times D\\ & +5.94\times C\times D+1.83\times A^{2}-0.17\times B^{2}-8.67\times C^{2}+3.83\times D^{2} \end{array}$$

#### 3.3.2. Parametric Influence on Thrust Force

**Figure 8:**
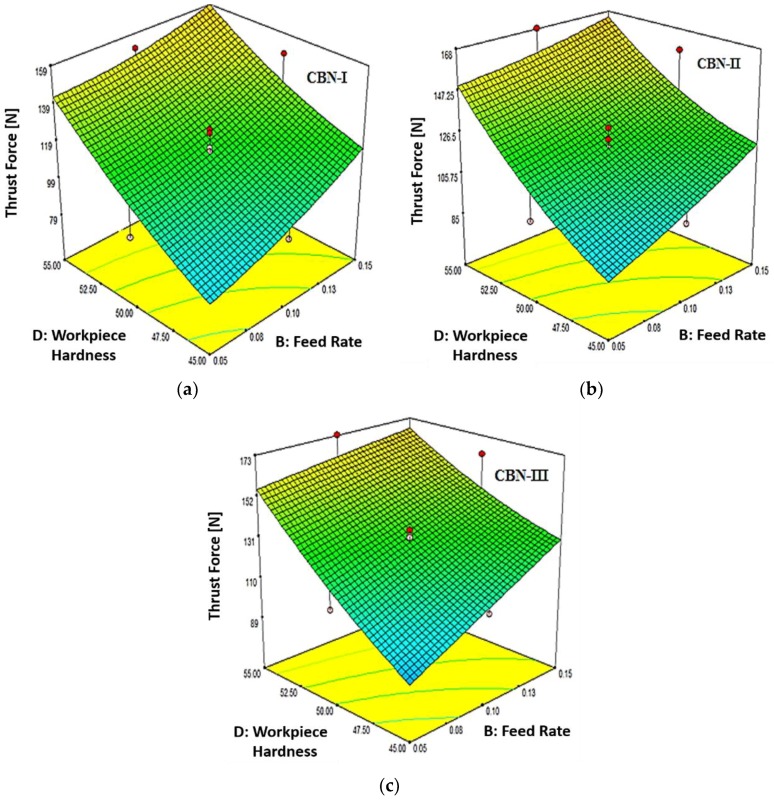
(**a**) Effect of Work Material Hardness (D) and Feed Rate (B) on Thrust Force (Ft) for CBN-I Insert; (**b**) Effect of Work Material Hardness (D) and Feed Rate (B) on Thrust Force (Ft) for CBN-II Insert; (**c**) Effect of Work Material Hardness (D) and Feed Rate (B) on Thrust Force (Ft) for CBN-III Insert.

**Figure 9:**
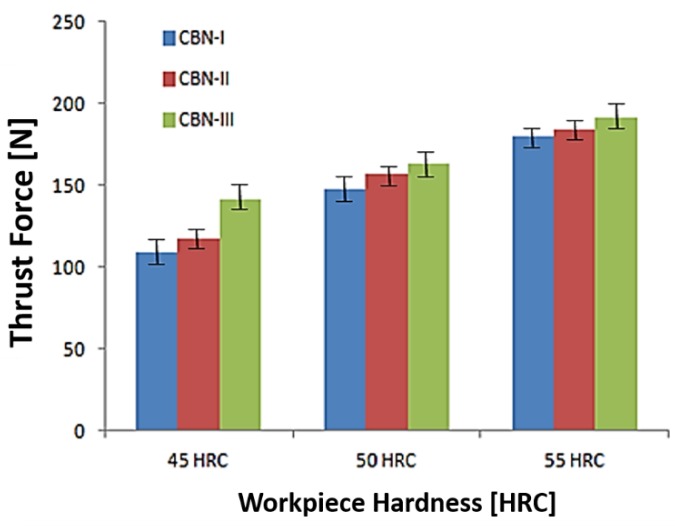
Comparisons of Thrust Force (Ft) with different CBN insert corresponding to workpiece Hardness.

In Figure 8a–c is presented the variation of thrust force corresponding to feed rate and workpiece hardness with the studied CBN-I, CBN-II and CBN-III inserts, respectively. Figure 9 revealed that thrust force increases with increase in material hardness and feed rate. It is highlighted that the higher workpiece hardness (55 HRC) shows the maximum thrust force with CBN-III insert. Further, it has been noticed that thrust force is found to be 30 to 70% higher than the tangential forces during hard turning of AISI H13 steel. 

From Figure 9 it has also been reported that CBN-III exhibit higher thrust force in a range of 68–141 N, 117–159 N and 123–191 N corresponding to material hardness of 45 HRC, 50 HRC and 55 HRC, respectively. While CBN-I exhibit a lower thrust force compare to other two CBN inserts at the similar machining conditions. 

### 3.4. Optimization of Cutting Conditions Using Desirability Approach

**Figure 10:**
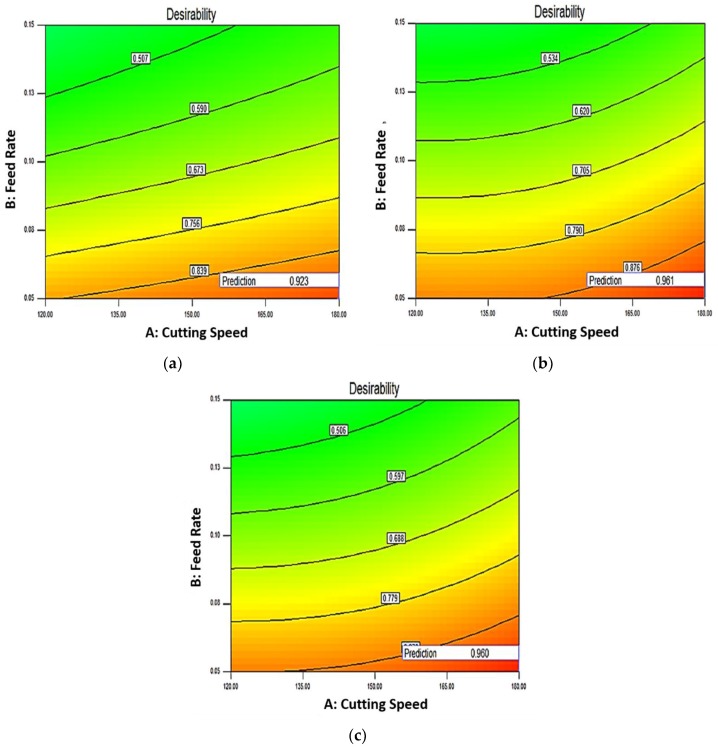
(**a**) Contour Plots of Desirability Function for CBN-I Inserts; (**b**) Contour Plots of Desirability Function for CBN-II Inserts; (**c**) Contour Plots of Desirability Function for CBN-III Inserts.

Desirability approach generally is used to carry out for the optimum parametric combinations for single and multiple optimizations [34,35,36]. This approach of optimization avoids the clashing of responses in respect of single response optimization. Table 14 depicts the weightage, importance and ranges of the parameters for optimization. Contour plot has been drawn to predict the overall desirability in the experimental domain at different zone for different CBN inserts. Figure 10a–c illustrate the contour plots of overall desirability for CBN-I, CBN-II and CBN-III inserts, respectively. Figure 10a shows that desirability for the CBN-I inserts gradually decreases, as we move for the left upward portion of the contour plots. The plot shows the optimum results at the bottom right side of the contour with desirability value of 0.92. Similarly Figure 10b,c illustrates the desirability value of 0.960 and 0.961 for CBN-II and CBN-III inserts, respectively. Table 15 represents the optimal solution and the desirability values for the hard turning of AISI H13 with different CBN inserts.

## 4. Conclusions

This study addresses the experimental results gathered from various CBN grades, of different hardness, in terms of cutting forces and surface roughness during hard turning of AISI H13 die tool steel. To find the optimal solution from multiple responses, desirability approach has been used. Following conclusions can be drawn from the above study:

1. This study demonstrated that CBN-I (BNX-10 Grade) insert is the best choice for continuous hard turning operation when compared to CBN-II (BN-600 Grade) and CBN-III (BNC-300 Grade) inserts. CBN-I generates a better surface roughness (*R_a_*) and lower cutting forces during the hard turning of AISI H13 die tool steel in respect to CBN-II (BN-600 Grade) and CBN-III (BNC-300 Grade) inserts that correspond to workpiece hardness of 45 HRC, 50 HRC and 55 HRC, respectively. Therefore, CBN-I (BNX-10 Grade) is recommended for continuous hard turning in order to obtain a better surface roughness (*R_a_*).
2. Surface roughness (*R_a_*) generated by CBN-I (BNX-10 Grade) insert are 26% and 36% better than CBN-II (BN-600 Grade) and CBN-III (BNC-300 Grade) inserts respectively from a workpiece having a hardness of 45 HRC. Similarly, CBN-I (BNX-10 Grade) depicts a better surface roughness (*R_a_*) of 14% and 32% at 50 HRC and 7% and 26% at 55 HRC.
3. The results revealed that the surface roughness (*R_a_*) decreases with the increase in workpiece hardness. Better surface roughness has been achieved at the higher hardness (55 HRC) as compare to lower workpiece hardness (50 HRC and 45 HRC), which confirm the potential of hard turning with CBN tool. Also surface roughness (*R_a_*) increases with increase in feed rate.
4. The tangential force (*F_c_*) is found to be maximum at higher workpiece hardness (55 HRC) with CBN-III (BNC-300 Grade) inserts, and decreases with workpiece hardness.
5. Thrust forces (*F_t_*) are 40–70% higher than the tangential forces and are found to be higher at the workpiece hardness of 55 HRC for each CBN inserts. It has also been noticed that CBN-III (BNC-300 Grade) insert produce the higher thrust force (*F_t_*) during hard turning, as compared to CBN-II (BN-600 Grade) and CBN-I inserts.
6. An optimum value of cutting conditions has been achieved with desirability function for CBN-I, CBN-II (BN-600 Grade) and CBN-III (BNC-300 Grade) inserts. The optimum cutting conditions for surface roughness, tangential force and thrust force corresponding to CBN-I (BNX-10 Grade) inserts are as cutting speed = 180 m/min, DOC = 0.08 mm, feed rate = 0.05 mm/rev, and workpiece hardness = 45 HRC.

## Figures and Tables

**Table 1:** Chemical Composition (%) of AISI H-13.

C	Mn	Si	Cr	Ni	Mo	V	Cu	P	S	Al	W	Fe
0.35%	0.32%	0.87%	5.04%	0.12%	1.64%	1.05%	0.19%	0.01%	0.00%	0.006%	0.01%	90.31%

**Table 2:** Properties of Different CBN Inserts.

CBN Inserts Type	CBN-I	CBN-II	CBN-III
Company Make	Sumitomo	Sumitomo	Sumitomo
Insert Grade	BNX 10	BN 600	BNC 300
Type	Low-CBN	High- CBN	Coated- CBN (TiAlN Coating)
Grain Binder	TiCN	Co-Al	TiN
CBN Content (%)	40–44	70–90	60–65
Hardness of Base material (HV)	2800–3000	3900–4200	
CBN Grain Size (μm)	0.5–1.0	1.8–2	1.0
Cutting Edge geometry	30° × 0.1 mm (Chamfered)	30° × 0.1 mm (Chamfered)	25° × 0.005 mm (Chamfered)

**Table 3:** Machining Parameters and their Levels.

Serial Number	Parameters (Unit)	Level-1	Level-2	Level-3
1	Cutting Speed, A (m/min)	120	150	180
2	Feed Rate, B (mm/rev)	0.05	0.10	0.15
3	Depth of Cut, C (mm)	0.08	0.13	0.18
4	Workpiece Hardness, D (HRC)	45	50	55

**Table 4:** Design Matrix and Results.

Run	C/S (m/min)	F/R (mm/rev)	DOC (mm)	W/P Hardness (HRC)	Surface Roughness (μm)			Tangential Force (N)			Thrust Force (N)		
					CBN-I	CBN-II	CBN-III	CBN-I	CBN-II	CBN-III	CBN-I	CBN-II	CBN-III
1	120	0.15	0.18	45	1.2	1.64	1.89	69	77	90	104	117	131
2	150	0.1	0.13	50	0.47	0.48	0.64	76	81	83	113	110	121
3	150	0.1	0.13	50	0.5	0.52	0.66	79	81	87	114	114	127
4	120	0.1	0.13	50	0.57	0.67	0.83	79	84	89	129	147	159
5	180	0.05	0.08	45	0.37	0.42	0.51	26	31	44	51	58	68
6	180	0.05	0.18	45	0.53	0.57	0.63	47	51	73	77	91	101
7	120	0.15	0.18	55	0.62	0.67	0.71	92	101	123	180	184	191
8	150	0.1	0.13	50	0.49	0.58	0.78	80	82	88	112	119	121
9	120	0.05	0.08	45	0.46	0.49	0.71	32	37	46	57	62	72
10	150	0.1	0.13	50	0.49	0.62	0.8	72	77	82	116	116	131
11	150	0.05	0.13	50	0.29	0.48	0.51	56	63	77	90	97	109
12	150	0.1	0.13	50	0.58	0.64	0.84	80	87	82	124	123	132
13	120	0.05	0.18	45	0.68	0.71	0.83	49	56	67	89	94	98
14	180	0.15	0.08	55	0.4	0.41	0.49	71	74	89	109	114	123
15	180	0.05	0.18	55	0.19	0.21	0.29	87	84	101	148	154	167
16	180	0.1	0.13	50	0.24	0.21	0.32	69	67	78	103	97	117
17	150	0.1	0.13	55	0.34	0.42	0.53	90	96	117	151	168	173
18	180	0.05	0.08	55	0.14	0.21	0.23	61	62	67	100	109	119
19	120	0.05	0.18	55	0.29	0.34	0.48	90	89	109	167	172	179
20	150	0.1	0.13	50	0.59	0.7	0.88	71	73	82	126	129	135
21	150	0.1	0.13	45	0.64	0.74	0.88	59	62	78	89	96	107
22	150	0.1	0.18	50	0.47	0.53	0.67	78	82	96	103	137	151
23	150	0.15	0.13	50	0.38	0.43	0.59	88	90	99	148	157	163
24	120	0.15	0.08	55	0.53	0.62	0.84	77	84	99	114	127	134
25	180	0.15	0.08	45	0.77	1.12	1.4	55	58	70	96	99	102
26	150	0.1	0.08	50	0.37	0.54	0.69	63	69	82	99	96	104
27	120	0.15	0.08	45	0.96	1.31	1.71	61	67	76	94	102	114
28	180	0.15	0.18	45	0.87	1.36	1.51	66	81	92	109	111	141
29	120	0.05	0.08	55	0.22	0.27	0.34	64	72	84	107	119	132
30	180	0.15	0.18	55	0.47	0.53	0.61	87	99	104	167	171	181

**Table 5:** ANOVA model of Surface Roughness for CBN-I Insert.

Source	Sum of Squares	Df	Mean Square	*F* Value	*p*-Value, Prob > F	Remarks
Model	1.42	14	0.1	12.01	<0.0001	significant
A-Cutting Speed	0.13	1	0.13	15.81	0.0012	
B-Feed Rate	0.51	1	0.51	60.4	<0.0001	
C-Depth of Cut	0.067	1	0.067	7.96	0.0129	
D-Workpiece Hardness	0.6	1	0.6	70.78	<0.0001	
Residual	0.13	15	8.44 × 10^−3^			
Lack of Fit	0.11	10	0.011	4.3	0.0606	not significant
Pure Error	0.013	5	2.64 × 10^−3^			
Cor Total	1.55	29				
Std. Dev.	0.092			R-Squared		0.9181
Mean	0.5			Adj R-Squared		0.8417
C.V. %	18.23			Pred R-Squared		0.717
PRESS	0.44			Adeq Precision		15.322

Degree of Freedom: Df; Std. Dev.: Standard Deviation; C.V. %: Coefficient of Variation %.

**Table 6:** ANOVA model of Surface Roughness for CBN-II Insert.

Source	Sum of Squares	Df	Mean Square	*F* Value	*p*-Value, Prob > F	Remarks
Model	2.98	14	0.21	9.64	<0.0001	Significant
A-Cutting Speed	0.16	1	0.16	7.12	0.0176	
B-Feed Rate	1.07	1	1.07	48.59	<0.0001	
D-Workpiece Hardness	1.22	1	1.22	55.22	<0.0001	
BD	0.26	1	0.26	11.8	0.0037	
Residual	0.33	15	0.022			
Lack of Fit	0.3	10	0.03	4.57	0.0537	not significant
Pure Error	0.033	5	6.52 × 10^−3^			
Cor Total	3.31	29				
Std. Dev.	0.15			R-Squared		0.9
Mean	0.61			Adj R-Squared		0.8067
C.V. %	24.15			Pred R-Squared		0.6176
PRESS	1.26			Adeq Precision		12.761

**Table 7:** ANOVA model of Surface Roughness for CBN-III Insert.

Source	Sum of Squares	Df	Mean Square	*F* Value	*p*-Value, Prob > F	Remarks
Model	4.12	14	0.29	11.12	<0.0001	significant
A-Cutting Speed	0.31	1	0.31	11.6	0.0039	
B-Feed Rate	1.51	1	1.51	57.23	<0.0001	
D-Workpiece Hardness	1.71	1	1.71	64.69	<0.0001	
BD	0.4	1	0.4	15	0.0015	
Residual	0.4	15	0.026			
Lack of Fit	0.35	10	0.035	3.73	0.0797	not significant
Pure Error	0.047	5	9.39 × 10^−3^			
Cor Total	4.52	29				
Std. Dev.	0.16			R-Squared		0.9121
Mean	0.76			Adj R-Squared		0.8302
C.V. %	21.4			Pred R-Squared		0.6671
PRESS	1.5			Adeq Precision		13.352

**Table 8:** ANOVA model of Tangential Force for CBN-I Insert.

Source	Sum of Squares	Df	Mean Square	*F* Value	*p*-Value, Prob > F	Remarks
Model	7604.28	14	543.16	34.94	<0.0001	significant
A-Cutting Speed	107.56	1	107.56	6.92	0.0189	
B-Feed Rate	1317.56	1	1317.56	84.75	<0.0001	
C-Depth of Cut	1334.72	1	1334.72	85.86	<0.0001	
D-Workpiece Hardness	3612.5	1	3612.5	232.38	<0.0001	
BC	100	1	100	6.43	0.0228	
BD	324	1	324	20.84	0.0004	
Residual	233.18	15	15.55			
Lack of Fit	151.85	10	15.19	0.93	0.5689	not significant
Pure Error	81.33	5	16.27			
Cor Total	7837.47	29				
Std. Dev.	3.94			R-Squared		0.9702
Mean	69.13			Adj R-Squared		0.9425
C.V. %	5.7			Pred R-Squared		0.9021
PRESS	767.16			Adeq Precision		24.231

**Table 9:** Model of Tangential Force for CBN-II Insert.

Source	Sum of Squares	Df	Mean Square	*F* Value	*p*-Value, Prob > F	Remarks
Model	7673.49	14	548.11	30.31	<0.0001	significant
A-Cutting Speed	200	1	200	11.06	0.0046	
B-Feed Rate	1922	1	1922	106.3	<0.0001	
C-Depth of Cut	1530.89	1	1530.89	84.67	<0.0001	
D-Workpiece Hardness	3226.72	1	3226.72	178.46	<0.0001	
BD	203.06	1	203.06	11.23	0.0044	
Residual	271.21	15	18.08			
Lack of Fit	158.37	10	15.84	0.7	0.7043	not significant
Pure Error	112.83	5	22.57			
Cor Total	7944.7	29				
Std. Dev.	4.25			R-Squared		0.9659
Mean	73.9			Adj R-Squared		0.934
C.V. %	5.75			Pred R-Squared		0.8843
PRESS	919.27			Adeq Precision		24.131

**Table 10:** Model of Tangential Force for CBN-III Insert.

Source	Sum of Squares	Df	Mean Square	*F* Value	*p*-Value, Prob > F	Remarks
Model	8444.64	14	603.19	25.79	<0.0001	significant
A-Cutting Speed	234.72	1	234.72	10.04	0.0064	
B-Feed Rate	1682	1	1682	71.92	<0.0001	
C-Depth of Cut	2178	1	2178	93.12	<0.0001	
D-Workpiece Hardness	3669.39	1	3669.39	156.89	<0.0001	
AD	182.25	1	182.25	7.79	0.0137	
BD	121	1	121	5.17	0.038	
CD	9	1	9	0.38	0.5444	
A2	121.6	1	121.6	5.2	0.0376	
D2	132.42	1	132.42	5.66	0.031	
Residual	350.83	15	23.39			
Lack of Fit	312.83	10	31.28	4.12	0.0659	not significant
Pure Error	38	5	7.6			
Cor Total	8795.47	29				
Std. Dev.	4.84			R-Squared		0.9601
Mean	85.13			Adj R-Squared		0.9229
C.V. %	5.68			Pred R-Squared		0.8373
PRESS	1431.06			Adeq Precision		22.549

**Table 11:** Model of Thrust Force for CBN-I Insert.

Source	Sum of Squares	Df	Mean Square	*F* Value	*p*-Value, Prob > F	Remarks
Model	24360.7	14	1740.05	15.06	<0.0001	significant
B-Feed Rate	3068.06	1	3068.06	26.56	0.0001	
C-Depth of Cut	5582.72	1	5582.72	48.33	<0.0001	
D-Workpiece Hardness	12640.5	1	12640.5	109.42	<0.0001	
CD	1425.06	1	1425.06	12.34	0.0031	
C^2^	546.06	1	546.06	4.73	0.0461	
Residual	1732.79	15	115.52			
Lack of Fit	1553.29	10	155.33	4.33	0.0598	not significant
Pure Error	179.5	5	35.9			
Cor Total	26093.5	29				
Std. Dev.	10.75			R-Squared		0.9336
Mean	112.87			Adj R-Squared		0.8716
C.V. %	9.52			Pred R-Squared		0.7013
PRESS	7793.4			Adeq Precision		16.228

**Table 12:** Model of Thrust Force for CBN-II Insert.

Source	Sum of Squares	Df	Mean Square	*F* Value	*p*-Value, Prob > F	Remarks
Model	25271.9	14	1805.13	12.24	<0.0001	significant
A-Cutting Speed	800	1	800	5.42	0.0343	
B-Feed Rate	2837.56	1	2837.56	19.24	0.0005	
C-Depth of Cut	6612.5	1	6612.5	44.82	<0.0001	
D-Workpiece Hardness	13230.2	1	13230.2	89.68	<0.0001	
CD	900	1	900	6.1	0.026	
Residual	2212.79	15	147.52			
Lack of Fit	1983.29	10	198.33	4.32	0.0599	not significant
Pure Error	229.5	5	45.9			
Cor Total	27484.7	29				
Std. Dev.	12.15			R-Squared		0.9195
Mean	119.67			Adj R-Squared		0.8443
C.V. %	10.15			Pred R-Squared		0.6575
PRESS	9412.33			Adeq Precision		15.253

**Table 13:** Model of Thrust Force for CBN-III Insert.

Source	Sum of Squares	Df	Mean Square	*F* Value	*p*-Value, Prob > F	Remarks
Model	25081	14	1791.5	16.08	<0.0001	significant
B-Feed Rate	3068.06	1	3068.06	27.53	<0.0001	
C-Depth of Cut	7688	1	7688	68.98	<0.0001	
D-Workpiece Hardness	12012.5	1	12012.5	107.79	<0.0001	
BD	855.56	1	855.56	7.68	0.0143	
CD	564.06	1	564.06	5.06	0.0399	
Residual	1671.68	15	111.45			
Lack of Fit	1498.85	10	149.88	4.34	0.0595	not significant
Pure Error	172.83	5	34.57			
Cor Total	26752.7	29				
Std. Dev.	10.56			R-Squared		0.9375
Mean	130.1			Adj R-Squared		0.8792
C.V. %	8.11			Pred R-Squared		0.7474
PRESS	6757.2			Adeq Precision		17.311

**Table 14:** Range of parameters with weight-age and importance for different inserts.

Parameters	Goal	CBN Grades	Lower Limit	Upper Limit	Lower Weight	Upper Weight	Imp
Cutting Speed (m/min)	is in range		120	180	1	1	3
Feed Rate (mm/rev)	is in range		0.05	0.15	1	1	3
Depth of Cut (mm)	is in range		0.08	0.18	1	1	3
Workpiece Hardness (HRC)	is in range		45	55	1	1	3
Surface Roughness (μm)	Minimize	CBN-I	0.14	1.2	1	1	3
		CBN-II	0.21	1.64	1	1	3
		CBN-III	0.23	1.89	1	1	3
Tangential Force (N)	Minimize	CBN-I	26	92	1	1	3
		CBN-II	31	101	1	1	3
		CBN-III	44	123	1	1	3
Thrust Force (N)	Minimize	CBN-I	51	180	1	1	3
		CBN-II	58	184	1	1	3
		CBN-III	68	191	1	1	3

**Table 15:** Desirability summary from results of different inserts.

Inserts	Cutting Speed (m/min)	Feed Rate (mm/rev)	Depth of Cut (mm)	Workpiece Hardness (HRC)	Surface Roughness (μm)	Tangential Force (F_c_) (N)	Thrust Force (F_t_) (N)	Desirability
**CBN-I**	180	0.05	0.08	45.27	0.34293	26.3473	54.0157	0.923
**CBN-II**	180	0.05	0.08	45.6	0.37019	31.0196	57.8928	0.961
**CBN-III**	180	0.05	0.08	45.98	0.41452	44.2859	67.999	0.96
